# A Rare Case of Disseminated Bacteroides fragilis Sternoclavicular Osteomyelitis Following Cardiopulmonary Resuscitation (CPR) in an Immunocompromised Patient: Is CPR the Missing Link?

**DOI:** 10.7759/cureus.99244

**Published:** 2025-12-15

**Authors:** Faiz Abdul Rahman, Hermine Carine Pouabe Epse Bodah, Manushyam Manimaran, Gayathri Rajmohan

**Affiliations:** 1 Geriatrics, Midland Metropolitan University Hospital, Birmingham, GBR; 2 General Internal Medicine, Midland Metropolitan University Hospital, Birmingham, GBR

**Keywords:** anaerobic infection, bacteroides fragilis, cardiopulmonary resuscitation, chest wall microtrauma, hematogenous spread, immunocompromised host, sternoclavicular osteomyelitis

## Abstract

Primary sternoclavicular osteomyelitis is uncommon and often misdiagnosed due to its nonspecific presentation, with anaerobic involvement, particularly by *Bacteroides fragilis*, being exceptionally rare and typically occurring in immunocompromised patients. We report the case of a 73-year-old man with multiple comorbidities, including diabetes mellitus, chronic kidney disease, ischaemic heart disease, and a chronic heel ulcer, who presented with fever and dyspnoea following a recent pulseless electrical activity arrest and successful cardiopulmonary resuscitation (CPR). Although initially managed for chronic calcaneal osteomyelitis, persistently elevated inflammatory markers and new anterior chest wall swelling prompted imaging that revealed a multiloculated abscess with sternoclavicular joint erosion, and blood cultures subsequently confirmed *B. fragilis*. Image-guided drainage and prolonged targeted antibiotic therapy resulted in both clinical and radiological improvement, allowing surgical intervention to be avoided given his high operative risk. This case underscores the importance of considering anaerobic pathogens in osteomyelitis among immunocompromised individuals and suggests a possible association between CPR-related microtrauma and subsequent sternoclavicular joint infection, highlighting the need for early multidisciplinary assessment and management.

## Introduction

Osteomyelitis is a serious bone infection that can lead to significant morbidity, particularly in immunocompromised individuals with comorbidities such as diabetes mellitus, chronic kidney disease, or intravenous drug use [1]. Sternoclavicular osteomyelitis (SCO) is very rare, representing less than 1% of all bone infection cases. Its presentation is often nonspecific and may mimic more common cardiopulmonary conditions, leading to delayed diagnosis [2]. The sternoclavicular joint is vulnerable to hematogenous seeding because of its rich venous plexus and limited soft tissue coverage, making even minor trauma a potential gateway for infection.

While *Staphylococcus aureus *is the most frequent causative organism in osteomyelitis, anaerobic bacteria, particularly *Bacteroides fragilis*, are uncommon and usually associated with intra-abdominal infections [3]. *B. fragilis* osteomyelitis at sites such as the sternoclavicular joint is exceedingly rare [4,5].

We present a case of primary SCO caused by *B. fragilis* in an elderly patient with multiple comorbidities, occurring after cardiopulmonary resuscitation (CPR). This report highlights the diagnostic challenges, explores the potential role of CPR-related microtrauma as a predisposing factor for sternoclavicular joint infection, and illustrates the effectiveness of conservative management in high-risk patients [6,7].

## Case presentation

A 73-year-old Asian man with an extensive medical history, including diabetes mellitus, hypertension, hyperlipidaemia, chronic kidney disease, ischaemic heart disease, heart failure with preserved ejection fraction (HFpEF), and alcohol dependence, presented with a one-day history of shortness of breath, productive cough, and fever. His past history was notable for a chronic left heel ulcer with underlying osteomyelitis and multiple toe amputations. Three weeks earlier, he had sustained an upper gastrointestinal bleed complicated by a pulseless electrical activity (PEA) cardiac arrest, requiring one cycle of CPR with full recovery.

On arrival, the patient was afebrile (temperature 36.8°C) and haemodynamically stable. Physical examination revealed a foul-smelling left heel ulcer measuring 7×8 cm. Laboratory studies demonstrated leukocytosis (white blood cell (WBC) 12.8×10⁹/L), elevated C-reactive protein (CRP) (159 mg/L), and anaemia (haemoglobin (Hb) 87 g/L). Given his diabetic foot history and recent imaging confirming left calcaneal osteomyelitis, he was treated empirically with intravenous flucloxacillin and clindamycin. Moreover, he received one unit of packed red cells for anaemia. Serial blood test results, including full blood count and CRP, are summarised in Table 1.

**Table 1:** Full blood count and CRP on admission, day 15, day 25, and day 39 M: male; F: female; CRP: C-reactive protein

Parameter	16/01/2025 (day 1)	31/01/2025 (day 15)	10/02/2025 (day 25)	24/02/2025 (day 39)	Reference range
White blood cell (×10⁹/L)	12.8	20.1	20.4	8.3	4.0-11.0
Haemoglobin (g/dL)	8.7	9.0	9.3	8.1	13.0-17.0 (M)/12.0-15.0 (F)
Neutrophils (×10⁹/L)	11.3	18.1	17.5	5.6	2.0-7.5
Platelets (×10⁹/L)	388	623	691	524	150-450
Creatinine (µmol/L)	126	169	116	168	60-110 (M)/45-90 (F)
CRP (mg/L)	159	388	116	39	<5

Blood cultures obtained on admission later grew *B. fragilis*. When this result became available on day 3, his antibiotics were escalated from flucloxacillin and clindamycin to intravenous piperacillin-tazobactam, with subsequent transition to oral co-trimoxazole and metronidazole based on susceptibility testing.

**Figure 1:**
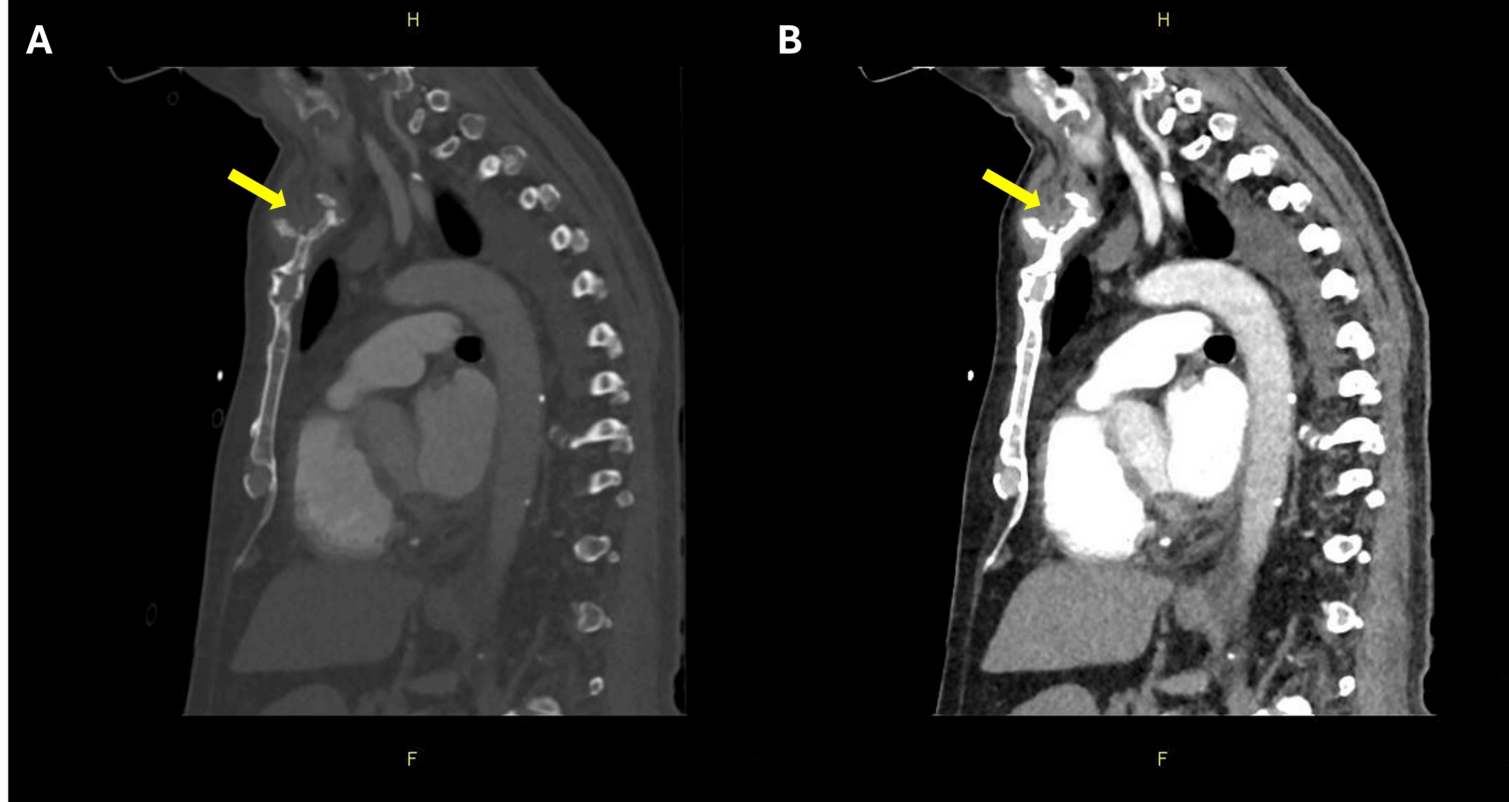
Sagittal CT images of the left sternoclavicular joint. (A) Soft tissue window demonstrating a multiloculated peri-sternoclavicular collection (yellow arrow). (B) Bone window of the same slice highlighting the cortical destruction of the medial clavicle and manubrium (yellow arrow), consistent with sternoclavicular osteomyelitis and septic arthritis

**Figure 2:**
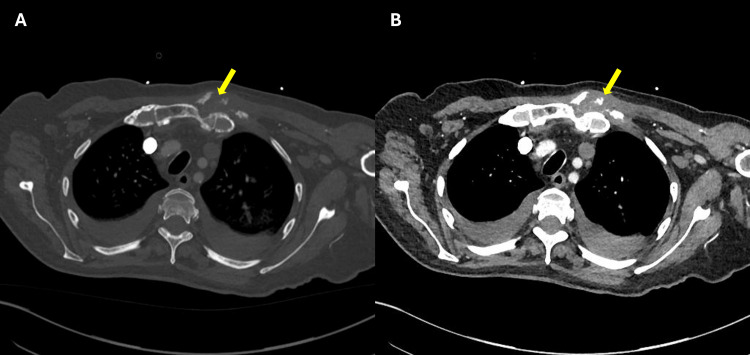
Axial CT images at the level of the left sternoclavicular joint. (A) Soft tissue window showing a large multiloculated collection extending into the anterior chest wall and adjacent muscle planes, centred at the sternoclavicular joint (yellow arrow). (B) Bone window of the same slice demonstrating the marked erosive destruction of the medial left clavicle and manubrium (yellow arrow), confirming septic arthritis with associated osteomyelitis

Two weeks later, the patient demonstrated worsening inflammatory markers (CRP 388 mg/L, WBC 20×10⁹/L) and developed new swelling over the left upper chest. The patient underwent CT imaging, which showed a large multiloculated fluid collection over the left sternoclavicular joint (11×6×5.6 cm) with bony erosions of the manubrium and clavicle, features consistent with destructive septic arthritis and osteomyelitis (Figures 1-2).

Given extensive comorbidities, surgical debridement was considered high risk, and a conservative management approach was favourable with prolonged targeted antibiotics.

Ultrasound-guided percutaneous drainage yielded purulent material, and a drain was left in situ for three weeks. No organisms were grown from fluid cultures; acid-fast bacilli staining and tuberculosis (TB) polymerase chain reaction (PCR) were negative, and histology excluded malignancy.

**Figure 3:**
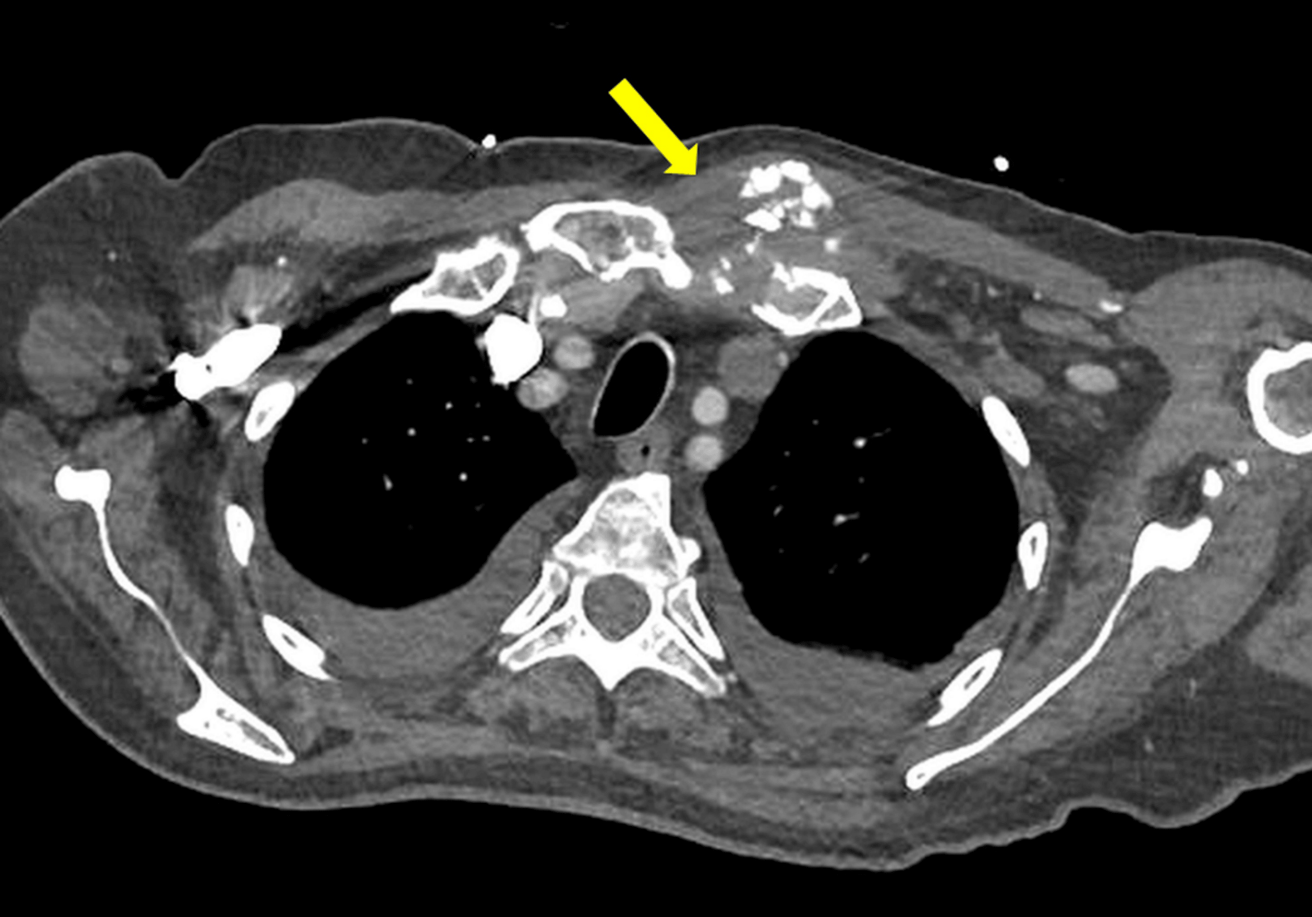
CT performed after treatment demonstrating the persistent bony destruction of the sternoclavicular joint (yellow arrow), which is expected following osteomyelitis, with no residual soft tissue collection

Follow-up CT three weeks later showed significantly reduced collection (4.5×2.2 cm) with persistent erosive changes. The patient was finally discharged with outpatient follow-up and an extended course of oral antibiotics. A further CT at three months showed the complete resolution of the soft tissue collection with expected residual bony destruction (Figure 3).

## Discussion

Osteomyelitis is a destructive infection of the bone that causes several complications if left untreated. SCO is particularly uncommon, accounting for less than 1% of all cases. Its clinical presentation often mimics cardiopulmonary pathologies, leading to diagnostic delays [1,2]. Risk factors include diabetes, chronic kidney disease, intravenous drug use, and immunosuppression [3].

Although *S. aureus* remains the most frequent cause of osteomyelitis, *B. fragilis*, a Gram-negative obligate anaerobe, is an extremely rare pathogen in bone infections. This organism is typically linked to intra-abdominal, pelvic, and soft tissue infections, with only a handful of cases reporting vertebral osteomyelitis caused by *B. fragilis* [3,4]. Park et al. demonstrated that anaerobic bacteria account for just 1.4% of vertebral osteomyelitis cases and *B. fragilis* were among the least common causes identified [3]. 

In this patient, several factors may have contributed to the development of infection, including uncontrolled diabetes, chronic kidney disease, and a chronic malodorous heel ulcer, which plausibly served as the primary source of bacteraemia. Haematogenous spread alone could fully account for the seeding of the sternoclavicular joint. The temporal proximity to recent CPR raises a theoretical possibility that CPR-related microtrauma might have created a locally susceptible environment; however, this remains speculative. Importantly, no imaging demonstrated haematoma, cartilage disruption, or any CPR-related injury. Therefore, CPR should be interpreted only as a potential, but unproven, contributing factor rather than an established cause. Only a few cases of SCO or mediastinal osteomyelitis following CPR have been reported, and none involve anaerobic SCO [8].

Diagnosis of SCO requires an integrated approach with a combination of clinical suspicion, imaging, and microbiology confirmation. Imaging modalities such as CT or MRI are often crucial for identifying bony erosion, joint effusion, and adjacent soft tissue involvement [1,9]. In this particular case, CT was pivotal in revealing radiological changes consistent with osteomyelitis, while ultrasound-guided drainage was essential for both diagnostic and therapeutic purposes. 

Although fluid cultures were negative, prior blood cultures identifying *B. fragilis* guided the choice of antibiotics. This highlights the importance of obtaining anaerobic cultures, as standard culture techniques may miss fastidious organisms [3,4].

Anaerobic osteomyelitis generally requires extended antibiotic therapy, given the difficulty of eradicating these organisms and the limited bone penetration of many antimicrobials [3,5]. Notably, *B. fragilis* has demonstrated increasing resistance to clindamycin and, in rare cases, metronidazole [4,10]. Broader analyses of sternoclavicular joint infections similarly report that these presentations remain uncommon, further underscoring the unusual nature of this case [11]. Therefore, blood culture sensitivities are fundamental to target therapy. Beta-lactam/beta-lactamase inhibitor combinations and carbapenems remain effective first-line options for resistant strains [4]. In this case, the patient responded well to a combination of co-trimoxazole and metronidazole, alongside image-guided drainage.

This treatment approach is consistent with current guidelines for managing SCO. Typically, a six-week course of antibiotics is recommended in the absence of prosthetic material, and longer durations are advised when the infection is extensive or the patient has other medical conditions [7]. While surgical debridement is often considered essential to prevent recurrence, in select cases like this one, where surgical risks are high, carefully monitored antibiotic therapy alone can be an effective alternative [2]. 

Managing resistance in *B. fragilis *infections requires awareness of emerging antimicrobial resistance patterns described in the broader literature. Although the isolate in this case was not tested for specific resistance mechanisms such as nim-gene-associated metronidazole resistance, these mechanisms have been reported and may influence treatment decisions in other settings [4]. Susceptibility testing remains essential to ensure the most appropriate antimicrobial is selected, and combination therapy may be required for severe or refractory infections [3,4].

The patient improved considerably with drainage and antibiotic treatment, as shown by a reduction in the size of the abscess on follow-up imaging. For complex cases like this, particularly those involving vital anatomical structures, a conservative approach is often the safest route. Continued follow-up through imaging and clinical assessment is essential to confirm that the infection is fully resolved and has not returned [1,2,7].

Key learning points

This case highlights several important learning points. CPR-related microtrauma may represent a previously under-recognised predisposing factor for sternoclavicular joint infection in susceptible patients. Anaerobic pathogens, although uncommon, should be considered in immunocompromised individuals or when atypical organisms are isolated. Additionally, in high-risk patients where surgery carries significant risk, conservative management with prolonged targeted antibiotics and image-guided drainage can be an effective therapeutic approach.

## Conclusions

This case highlights the exceptionally rare involvement of *B. fragilis* in SCO in a patient with several well-established risk factors, including diabetes mellitus, chronic kidney disease, alcohol dependence, and a chronic heel ulcer with underlying osteomyelitis. These comorbidities, particularly the chronic calcaneal osteomyelitis, provide a clear and plausible source of bacteraemia, making haematogenous seeding the most likely mechanism of sternoclavicular joint infection.

We propose that bacteraemia originating from chronic calcaneal osteomyelitis allowed *B. fragilis* to seed this traumatised region. Recognising CPR-related microinjury as a potential contributor to post-resuscitation infections may support earlier diagnosis and more tailored management in high-risk individuals. Further research is warranted to clarify this emerging association.
